# Neuroprotective Effects of Peanut Skin Extract Against Oxidative Injury in HT-22 Neuronal Cells

**DOI:** 10.3390/ph18040544

**Published:** 2025-04-08

**Authors:** Jinlan Huang, Yue Zhou, Hui Xu, Mingfu Wang

**Affiliations:** 1 Institute for Advanced Study, Shenzhen University, Shenzhen 518060, China; 2 College of Chemistry and Environmental Engineering, Shenzhen University, Shenzhen 518060, China; 3 School of Grain Science and Technology, Jiangsu University of Science and Technology, Zhenjiang 212000, China

**Keywords:** peanut skins, neuroprotection, antioxidant, PI3K/Akt, Nrf2

## Abstract

**Background:** Oxidative stress is a key therapeutic target in neurological disorders. As processing wastes from the peanut industry, peanut skins are great sources of antioxidants and possess potential in neuroprotection. **Methods:** We prepared a peanut skin extract (PSE) and investigated its protective effects against tert-butyl hydroperoxide (t-BHP)-induced oxidative injury in HT-22 neuronal cells. **Results:** PSE was rich in phenolic compounds (123.90 ± 0.46 mg GAE/g), comprising flavonoids (75.97 ± 0.23 mg RE/g) and proanthocyanidins (53.34 ± 1.58 mg PE/g), and displayed potent radical scavenging activities in chemical-based assays. In HT-22 cells, PSE pretreatment restored oxidative balance and endogenous antioxidant defense disrupted by t-BHP, as evidenced by significant reductions in ROS generation and lipid peroxidation levels, along with enhanced endogenous antioxidants. Specifically, 25 μg/mL PSE pretreatment reduced ROS levels by 53.03%, decreased MDA content by 78.82%, enhanced superoxide dismutase (SOD) activity by 12.42%, and improved the ratio of glutathione (GSH) to oxidized glutathione (GSSG) by 80.34% compared to the t-BHP group. Furthermore, PSE rescued mitochondrial membrane potential collapse, inhibited cytochrome c (Cyt.c) release, and prevented subsequent apoptotic death. Notably, the neuroprotective efficacy of PSE was comparable to that of edaravone, an approved neuroprotective drug. Mechanistic investigations combining network pharmacology and experimental validation revealed that the PI3K/Akt/Nrf2 signaling pathway played a pivotal role in mediating the neuroprotective effects of PSE. Compared to t-BHP-treated cells, 25 µg/mL PSE pretreatment significantly upregulated PI3K/Akt phosphorylation, the expression of Nrf2, and its downstream antioxidant proteins heme oxygenase-1 (HO-1) and NAD(P)H dehydrogenase quinone 1 (NQO1). **Conclusions:** Collectively, these findings demonstrate the potential of PSE as a natural protective agent against oxidative-related neurological disorders.

## 1. Introduction

With the global trend of population aging, the burden of central nervous system (CNS) disorders is on the rise, especially ischemic stroke, Alzheimer’s disease (AD), and Parkinson’s disease (PD) [1]. This phenomenon has become a major challenge for the global healthcare system in the 21st century. Currently, there are no pathophysiology-based cures for CNS disorders, and the prevailing therapeutic strategies remain largely limited to symptomatic management [2]. Brain injury in CNS disorders shares characteristic pathological processes, including oxidative stress, neuroinflammation, excitatory toxicity, apoptosis, aberrant ion channel function, and mitochondrial dysfunction, which collectively contribute to neuronal damage [3,4]. Therefore, neuroprotective agents targeting these pathological processes represent a promising strategy in the prevention and treatment of CNS disorders [5,6].

Oxidative stress and mitochondrial dysfunction are closely intertwined in the pathogenesis of CNS disorders. Mitochondria, as the primary producers of cellular energy via oxidative phosphorylation (OXPhos), are also significant sources of reactive oxygen species (ROS) due to electron leakage during the electron transport chain (ETC) process [7]. Under physiological conditions, these ROS are neutralized by intracellular antioxidant systems, such as glutathione (GSH), superoxide dismutase (SOD), and catalase (CAT), maintaining equilibrium between the generation of free radicals and antioxidant defenses [8]. However, this equilibrium is disrupted in CNS disorders. Uncontrolled oxidative stress contributes to mitochondrial dysfunction, which in turn produces more ROS [9]. The irreparable damage of mitochondria ultimately results in the release of pro-apoptotic factors, such as cytochrome c (Cyt.c), initiating cell apoptosis [10]. The Cyt.c-mediated mitochondrial-dependent apoptosis pathway is a critical mechanism for neuronal programmed cell death in response to oxidative stress. This process leads to a substantial loss of neurons, ultimately resulting in brain dysfunction and further contributing to CNS disorders [11]. Hence, oxidative stress is an important target, and antioxidants are a major focus of neuroprotection research. The PI3K/Akt (phosphatidylinositol 3-kinase/protein kinase B) signaling pathway is crucial in regulating multiple cellular processes related to CNS health, such as cell proliferation, mitochondrial function, and apoptosis. In addition, activated PI3K catalyzes the synthesis of phosphatidylinositol (3,4,5) trisphosphate (PIP3), which then activates Akt via 3-phosphoinositide-dependent protein kinase 1 (PDK1). Akt phosphorylates serine residues on nuclear factor erythroid 2-related factor 2 (Nrf2), promoting its translocation into the nucleus [12]. Nrf2 translocation upregulates antioxidant enzymes, which are vital for the elimination of ROS. Edaravone (Eda), a free radical scavenger, has been approved as a neuroprotective drug for the treatment of ischemic stroke and amyotrophic lateral sclerosis in Japan and the United States [13]. Our team has also identified several neuroprotective stilbenes, including pinosylvin and cajaninstilbene acid, which have the potential to mitigate cerebral ischemia-reperfusion injury through activation of the Nrf2 signaling pathway [14,15].

Peanut (*Arachis hypogaea* L.) skins are processing waste from the peanut industry, with an estimated annual global production of 740,000 metric tons [16]. Traditional Chinese medicine uses peanut skins to stop bleeding, disperse blood stasis, and reduce swelling [17]. Modern studies have shown that peanut skins are rich in phenolic compounds, including proanthocyanidins, flavanols, stilbenes, and phenolic acids, which provide beneficial bioactivities, particularly anti-oxidation and anti-inflammation [16,18,19]. Additionally, some phenolic constituents in peanut skins (e.g., fustin, glycitin, and oligomeric proanthocyanidins [20]) have exhibited neuroprotective properties, such as improving cognitive function and treating neurodegenerative disorders [16]. Nevertheless, the neuroprotective effects of peanut skins remain unexplored. The hippocampus plays a crucial role in maintaining brain homeostasis, and its impairment is strongly implicated in various CNS diseases [21]. Hence, this study aims to investigate the protective effects and mechanisms of the total polyphenol extract from peanut skins against oxidative stress-induced neuronal damage in HT-22 cells. We hope that this study will aid in the value-added use of peanut skins and the development of functional foods for neurological health.

## 2. Results

### 2.1. Peanut Skin Extract (PSE) Showed Antioxidant Activity in Chemical-Based Systems

PSE had a high content of phenolic compounds. The total phenolic content (TPC), total proanthocyanidin content (TPAC), and total flavonoid content (TFC) were 123.90 ± 0.46 mg (gallic acid equivalent, GAE)/g, 53.34 ± 1.58 mg (proanthocyanidin equivalent, PE)/g, and 75.97 ± 0.23 mg (rutin equivalent, RE)/g, respectively (Table 1), consistent with previously reported values [22,23]. Proanthocyanidins constitute the primary components of polyphenols in PSE. Given its high polyphenol content, the antioxidant ability of PSE was subsequently assessed using chemical-based assays, with ascorbic acid and Trolox used as reference antioxidants. As shown in Table 1, PSE exhibited potent free radical scavenging potential, with 2,2′-azino-bis (3-ethylbenzothiazoline-6-sulfonic acid) diammonium salt (ABTS), 2,2-diphenyl-1-picrylhydrazyl (DPPH), and hydroxyl radical scavenging activity (•OH-RSA) of 230.36 ± 15.96 mg (ascorbic acid equivalent, AAE)/g, 10.21 ± 0.12 mg (Trolox equivalent, TE)/g, and 136.49 ± 3.26 mg AAE/g, respectively. Additionally, the ferric reducing antioxidant power (FRAP) value of 14.59 ± 1.12 mg AAE/g (as shown in Table 1) indicated a relatively moderate reducing capacity of PSE.

### 2.2. PSE Prevented Tert-Butyl Hydroperoxide (t-BHP)-Induced Oxidative Stress in HT-22 Cells

**Figure 1:**
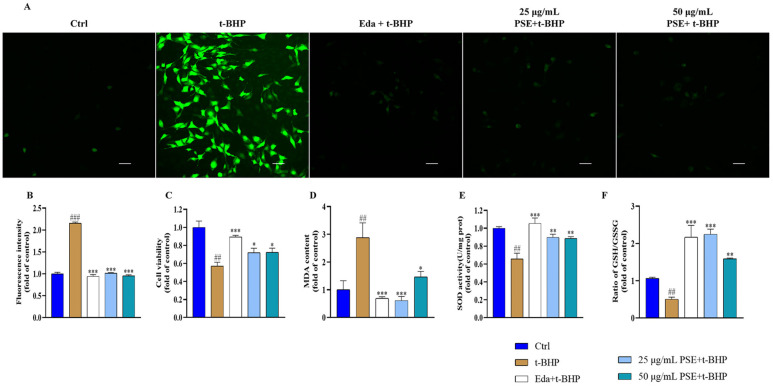
Antioxidant activity of PSE in HT-22 cells. (**A**) Intracellular ROS were stained using DCFH-DA and visualized under a fluorescence microscope (scale bar: 50 μm). (**B**) Quantitative analysis of the mean fluorescence density. (**C**) A CCK-8 assay was used to assess the effect of PSE on t-BHP-induced cytotoxicity in HT-22 cells. (**D**–**F**) Levels of MDA, SOD, and the ratio of GSH/GSSG in HT-22 cells were measured with the corresponding assay kits. All results were expressed as the means ± SD of at least three independent experiments. ^##^ *p* < 0.01, ^###^ *p* < 0.001 versus control; * *p* < 0.05, ** *p* < 0.01, *** *p* < 0.001 versus t-BHP group.

To further assess the antioxidant potential of PSE, we examined its protective effects against t-BHP-induced oxidative damage in HT-22 cells. As shown in Figure 1A,B, PSE pretreatment markedly attenuated t-BHP-induced intracellular ROS accumulation, as evidenced by the restoration of 2′,7′-dichlorodihydrofluorescein diacetate (DCFH-DA) fluorescence intensity to baseline levels. Furthermore, PSE pretreatment significantly improved HT-22 cell viability compared to the t-BHP group (Figure 1C). Malondialdehyde (MDA), another biomarker of oxidative stress, is a reliable indicator of lipid peroxidation levels. The levels of MDA in HT-22 cells increased in response to t-BHP treatment. Pre-treatment with 25 μg/mL PSE reduced MDA levels by 78.82% compared to the t-BHP group, while 8.71 μg/mL Eda achieved a 76.08% reduction, indicating comparable efficacy between PSE and Eda (Figure 1D). SOD and reduced GSH are key components of intracellular antioxidant defense. The SOD activities of the PSE-treated groups were significantly higher than those of the t-BHP group (Figure 1E). Figure 1F also demonstrates a markedly elevated GSH/oxidized glutathione (GSSG) ratio in the PSE group compared to the t-BHP group. Collectively, these results demonstrated that PSE exerted neuroprotective effects by preventing the oxidative stress induced by t-BHP.

### 2.3. PSE Attenuated t-BHP-Induced Mitochondrial Dysfunction and Apoptosis in HT-22 Cells

**Figure 2:**
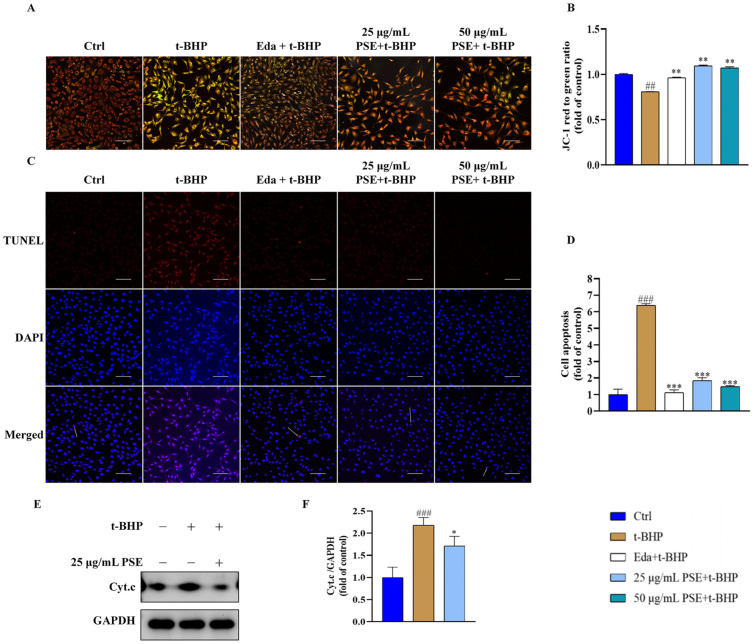
PSE inhibited mitochondrial dysfunction and apoptosis in t-BHP-damaged HT-22 cells. JC-1 staining was used to evaluate the ΔΨ_m_ of the HT-22 cells. (**A**,**B**) Images of JC-1-stained cells were captured using a fluorescence microscope (scale bar: 100 μm). The red signal represents normal ΔΨ_m_, while the green signal represents decreased ΔΨ_m_, and quantitative analysis of the ratio of red to green fluorescence intensity. (**C**,**D**) Apoptosis of HT-22 cells was examined using TUNEL staining. Images were captured using a confocal laser scanning microscope (CLSM) (scale bar: 100 μm) and quantitatively analyzed using Image J software (×64). (**E**,**F**) Western blotting was used to analyze the expression level of Cyt.c with glyceraldehyde-3-phosphate dehydrogenase (GAPDH) as the internal control. All results were expressed as means ± SD of at least three independent experiments. ^##^ *p* < 0.01, ^###^ *p* < 0.001 versus control; * *p* < 0.05, ** *p* < 0.01, *** *p* < 0.001 versus t-BHP group.

Mitochondria generate ATP (Adenosine triphosphate) by harnessing electrochemical gradients across the inner mitochondrial membrane (negative inside) established by the ETC. Sustained/excess oxidative stress compromises mitochondrial membrane potential due to mechanisms such as ETC impairment and increased membrane permeability. The depolarization leads to mitochondrial dysfunction, further promoting ROS production and apoptosis activation [24]. Therefore, the impact of PSE on mitochondrial function in HT-22 cells was evaluated by monitoring mitochondrial membrane potential (ΔΨm) changes using a 5,5′,6,6′-tetrachloro-1,1′,3,3′-tetraethyl-imidacarbocyanine (JC-1) fluorescent probe. The probe emits red fluorescence at normal ΔΨ_m_ and green at low ΔΨ_m_. As shown in Figure 2A,B, t-BHP-treated cells exhibited a decreased red/green fluorescence ratio (ΔΨ_m_ = 0.757 ± 0.003), indicating mitochondrial membrane depolarization. In contrast, PSE pretreatment restored ΔΨ_m_, as shown by the increased ratio of red/green fluorescence (ΔΨ_m_ = 1.094 ± 0.009). Mitochondrial dysfunction triggers the release of many pro-apoptotic factors, such as Cyt.c. The release of Cyt.c from the mitochondria initiates the caspase cascade, leading to apoptotic cell death. Western blot analysis demonstrated that pretreatment of cells with PSE inhibited Cyt.c release, as evidenced by the reduction of Cyt.c proteins in PSE-treated cells compared to that of t-BHP-treated cells (Figure 2E,F). Furthermore, TdT-mediated dUTP nick-end labeling (TUNEL) staining reflected significantly increased apoptosis in HT-22 cells in response to t-BHP treatment, which was consistent with the loss of cell viability. In contrast, cells pretreated with PSE showed a significantly decreased apoptotic rate compared to the t-BHP group (Figure 2C,D), suggesting that PSE improved cell survival by inhibiting apoptotic death. Notably, the protective effects of PSE were comparable to those of Eda. These findings highlight the neuroprotective efficacy of PSE by inhibiting mitochondrial dysfunction and subsequent apoptotic death in cells injured by t-BHP.

### 2.4. Network Pharmacological Analysis of the Neuroprotective Mechanisms and Targets of PSE

**Figure 3:**
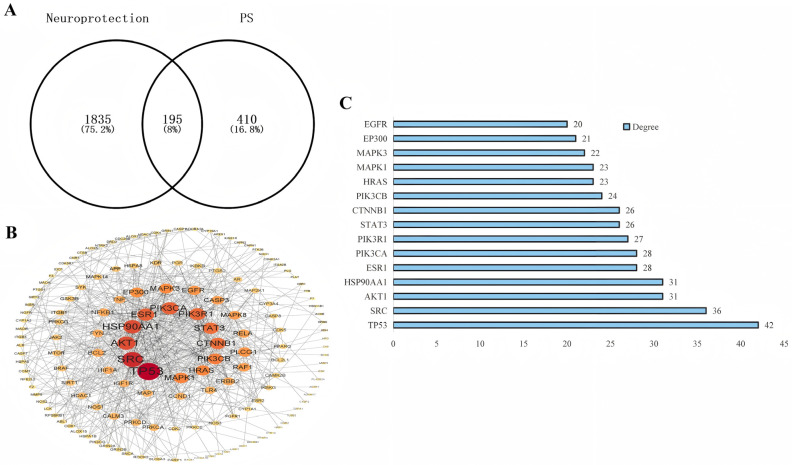
Network pharmacological analysis of the potential neuroprotective targets of PSE. (**A**) Venn diagram of the overlapping targets between the targets of peanut skin (PS) phenolic compounds and neuroprotection-related targets. (**B**) PPI network of 195 overlapping targets. The larger the red circle and font size, the greater the degree of connection within the PPI network. (**C**) The top 15 targets with the highest degree of connection in the PPI network.

Network pharmacology enables prediction of potential molecular mechanisms through construction of a multi-dimensional “compound-target-pathway” network. The analysis can inform subsequent experimental designs and enhance research efficiency. Therefore, we employed network pharmacology to explore the neuroprotective mechanism of PSE. First, we compiled compound targets of peanut skin polyphenols and neuroprotection-related disease targets. Through a comprehensive literature review (Table 2) [25,26,27], we identified 17 principal phenolic constituents in peanut skins, including six phenolic acids, eight flavonoids, and three stilbenes. Based on these compounds, a total of 605 potential molecular targets were predicted using the SwissTargetPrediction database. Additionally, we identified 2030 neuroprotection-related targets from five major databases (GeneCards, Online Mendelian Inheritance in Man database (OMIM), Therapeutic Target Database (TTD), PharmGKB, and DrugBank). Next, a Venn diagram was utilized to analyze the common targets, followed by protein–protein interaction (PPI) analysis to identify hub targets. The Venn diagram (Figure 3A) revealed 195 overlapping targets. The PPI networks of these 195 targets were then constructed using the STRING database and Cytoscape software (3.9.1). The resulting network contained 150 nodes and 631 edges (Figure 3B). Using the CytoNAC plugin, we identified the top 15 targets based on interaction degree (Figure 3C). These targets likely constitute the core mediators of PSE’s neuroprotective effects. Finally, we performed functional characterization through Gene Ontology (GO) biological function enrichment and Kyoto Encyclopedia of Genes and Genomes (KEGG) pathway analyses.

**Figure 4:**
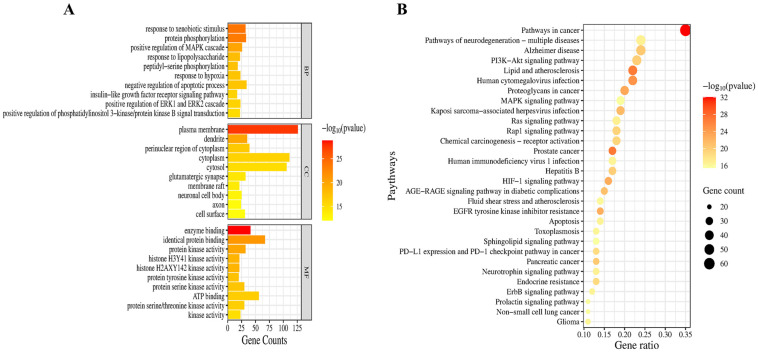
GO and KEGG enrichment analysis. (**A**) GO enrichment analysis was conducted, revealing the top 30 GO functional items based on their *p*-values. In the histogram, the ordinate represents the GO term, while the abscissa indicates the number of genes associated with each term. The length of each bar signifies the number of targets within each GO term. (**B**) KEGG enrichment analysis was performed. In the bubble chart, the ordinate represents the KEGG term, while the abscissa signifies the proportion of targets associated with each term. The size of each circle indicates the number of enriched targets within the specific KEGG term. The color of the circles represents the *p*-value, with a redder color indicating a higher degree of enrichment and a correspondingly smaller *p*-value.

The top GO functional items are presented in Figure 4A. The results showed that PSE may exert neuroprotection against oxidative stress through a series of biological processes, including positive regulation of the mitogen-activated protein kinase (MAPK), extracellular-regulated kinase 1/2 (ERK1/2) and PI3K/Akt cascades as well as negative regulation of the apoptotic process. KEGG analysis identified 184 pathways related to the neuroprotective effects of PSE, and the top 30 most significant pathways are listed in Figure 4B. Among the enriched pathways, the PI3K/Akt pathway emerged as the most prominent mechanism underlying the neuroprotective effects of PSE, as evidenced by its significant *p*-value (1.58 × 10^−20^), and the largest number of associated genes (Gene counts = 43). In addition, the pathway is an important regulator of multiple processes affected by PSE, including oxidative stress, mitochondrial dysfunction, and apoptosis, as demonstrated in our experimental results. It is also a promising therapeutic target for major CNS disorders, like ischemic stroke, PD, and AD [28]. Therefore, we focused on mechanistic investigation of the PI3K/Akt pathway.

### 2.5. The PSE Upregulated PI3K/Akt/Nrf2 Pathway in HT-22 Cells

**Figure 5:**
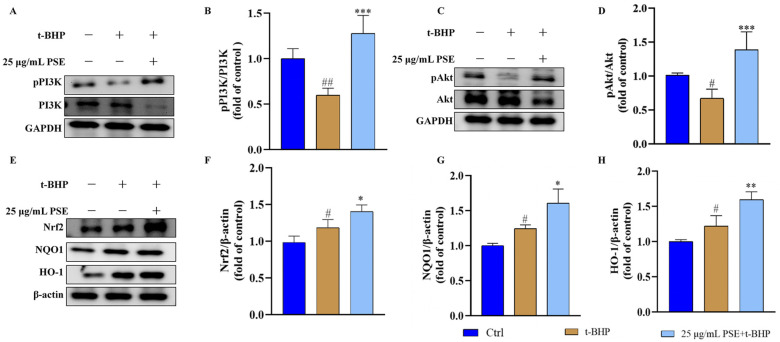
PSE activated the PI3K/Akt/Nrf2 pathway in HT-22 cells. (**A**,**C**,**E**) Western blot was used to detect the expression levels of PI3K, pPI3K, Akt, pAkt, Nrf2, NQO1, and HO-1 in total proteins from HT-22 cells, with GAPDH and β-actin as the internal control separately. (**B**,**D**) Quantitative analysis of the ratio of pPI3K/PI3K and pAkt/Akt in different groups. (**F**–**H**) Quantitative analysis of the expression levels of Nrf2, NQO1, and HO-1 in different groups. All results were expressed as means ± SD of at least three independent experiments. ^#^ *p* < 0.05, ^##^ *p* < 0.01 versus control; * *p* < 0.05, ** *p* < 0.01, *** *p* < 0.001 versus t-BHP group.

The PI3K/Akt pathway is a crucial regulator of cell proliferation, anti-apoptosis, and cellular defense [28]. As shown in Figure 5A,B, the t-BHP group exhibited lower levels of phosphorylated PI3K (pPI3K)/PI3K than the control group. In contrast, PSE-pretreated cells displayed significantly enhanced PI3K phosphorylation levels when challenged with t-BHP. Likewise, PSE treatment resulted in the induction of Akt phosphorylation, with the ratio of phosphorylated Akt (pAkt)/Akt being 56.48% higher than that of the t-BHP treatment group (Figure 5C,D). Clearly, PSE promotes the activation of the PI3K/Akt pathway in HT-22 cells, representing a key mechanism by which PSE exerts its neuroprotective effects.

Nrf2, a master transcriptional regulator of cellular antioxidant responses, functions downstream of the PI3K/Akt signaling pathway. To explore whether PSE exerts its antioxidant activity by activating the Nrf2 pathway, key components of the Nrf2 pathway were examined. Nrf2 resides in the cytoplasm, under physiological conditions. However, oxidative stress promotes its nuclear translocation, where it binds to the antioxidant response element (ARE) in the promoter regions of target genes, including heme oxygenase-1 (HO-1) and NAD(P)H dehydrogenase quinone 1 (NQO1) [29]. As shown in Figure 5E–H, after 24 h of treatment with 25 µg/mL PSE, the protein levels of total Nrf2, HO-1, and NQO1 in HT-22 cells were upregulated, suggesting activation of the Nrf2 pathway. In summary, the data demonstrated that PSE exerted protective effects against oxidative stress in HT-22 cells through the activation of the PI3K/Akt/Nrf2 pathway.

## 3. Discussion

The brain requires a substantial amount of energy to sustain neuronal viability and physiological functions. This energy primarily relies on aerobic respiration, an oxygen-intensive metabolic process that renders the brain particularly vulnerable to oxidative stress. In CNS disorders, oxidative stress is a key event that precedes multiple pathological processes, including mitochondrial dysfunction, apoptosis, and inflammation [30,31]. The principal objective of neuroprotective strategies is to preserve neuronal survival and prevent secondary injuries caused by oxidative stress and inflammatory responses. Studies have shown the potential of phenolic compounds in alleviating neurological disorders, primarily through their antioxidant properties [32]. The present study found that PSE contains a high concentration of phenolic compounds, particularly in proanthocyanidins and flavonoids. Consequently, PSE exhibited potent free radical scavenging capacity, as assessed by the ABTS, DPPH, and hydroxyl radical scavenging assays. To examine the neuroprotective potential of PSE, we established an in vitro model using t-BHP-challenged HT-22 mouse hippocampal neuron cells. Previous reports have demonstrated that the cell line exhibited highly consistent experimental results when compared to human neurons [33]. This model is well suited for studies of oxidative stress-induced neurotoxicity and has been widely used in research pertaining to neurological disorders [34]. Following stimulation with t-BHP, HT-22 cells displayed marked oxidative stress damage, as evidenced by elevated levels of ROS and MDA, along with reduced cellular antioxidants GSH and SOD. Treatment with 25 μg/mL of PSE demonstrated significant antioxidant effects, comparable to those of 8.71 μg/mL of Eda. Both treatments also markedly improved the GSH/GSSG ratio, with 8.71 μg/mL of Eda enhancing it by 73.67% and 25 μg/mL of PSE improving it by 80.34%. The reduced oxidative stress prevented further cellular damage and promoted cell viability.

Mitochondria are vulnerable to oxidative stress-induced damage. Mitochondrial dysfunction has been implicated in the pathogenesis of various CNS disorders [35]. Excess oxidative stress could promote mitochondrial depolarization, resulting in the collapse of ΔΨm. This increases free radical production and compromises antioxidant defense, ultimately leading to the release of mitochondrial proteins. Among these proteins, Cyt.C is critical due to its role in initiating the intrinsic apoptotic pathway [36]. Therefore, maintaining mitochondrial integrity against oxidative stress is essential for neuronal survival. In this study, we observed that cells treated with PSE retained normal ΔΨ_m_ when challenged with t-BHP, as evidenced by JC-1 staining. Furthermore, Western blot analysis showed reduced Cyt. c levels in the PSE-treated cells compared to the t-BHP-treated group. These findings suggest that PSE effectively alleviates t-BHP-induced mitochondrial dysfunction. Consequently, PSE treatment decreased the apoptotic rate and improved cell survival. During oxidative stress, Nrf2 pathway activation attenuates mitochondrial dysfunction by upregulating antioxidant defenses [37]. Specifically, this pathway enhances the expression of mitochondrial-specific antioxidant enzymes, including an increase in the expression of SOD and glutathione peroxidases (GPx), which effectively sustain the levels of antioxidant enzymes within the mitochondria and facilitate the clearance of ROS [38,39]. Additionally, the upregulation of NQO1 expression strengthens the mitochondrial antioxidant defense capacity [40], while the increased expression of HO-1 promotes ATP production by enhancing mitochondrial biogenesis and energy metabolism [41]. Collectively, these synergistic effects contribute to the maintenance of mitochondrial integrity.

**Figure 6:**
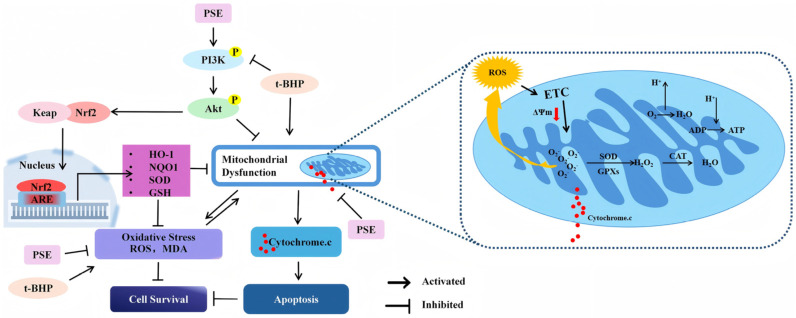
Neuroprotective mechanisms of PSE against t-BHP-induced oxidative stress. PSE quenched the free radicals generated in response to t-BHP treatment. Meanwhile, PSE enhanced the phosphorylation of PI3K and Akt, which activated Nrf2, leading to an upregulation of the antioxidant system. This process improved cellular resistance to oxidative stress. Notably, this pathway specifically enhances the expression of mitochondrial-specific antioxidant enzymes that help eliminate ROS. Additionally, phosphorylated Akt can directly influence mitochondrial activity and prevent mitochondrial dysfunction.

Network pharmacology is a powerful tool for exploring interactions between pharmacological compounds and disease targets, facilitating the identification of molecular mechanisms. The findings from the integrated analysis of PPI, KEGG, and GO enrichment indicated the PI3K/Akt signaling pathway as the major pathway affected by PSE, with Akt as a key target. The PI3K/Akt signaling pathway is essential in regulating multiple cellular processes associated with CNS development and function. These processes encompass cell survival, autophagy, neurogenesis, neuronal proliferation and differentiation, and synaptic plasticity [42]. Nrf2, a key transcription factor and downstream effector in the PI3K/Akt pathway, is a master regulator of endogenous antioxidant defense. Studies have shown that PI3K/Akt activation could promote Nrf2 nuclear translocation, thus enhancing the expression of antioxidant enzymes (e.g., NQO1 and HO-1) to inhibit oxidative damages [12]. In addition, Akt could regulate the B-cell lymphoma-2 (Bcl-2) family proteins and mitochondrial membrane permeability, preventing the release of Cyt.c and other pro-apoptotic factors from the mitochondrial intermembrane space [43,44,45]. As reported by Bai Gao el., the activated AKT signaling pathway alleviated mitochondrial dysfunction and oxidative stress to reduce neuronal damage [46]. Besides, previous research has demonstrated that proanthocyanidins, the major phenolic constituents identified in PSE in this study, could activate the PI3K/Akt and Nrf2 signaling pathways [47]. To validate the results of the pharmacological analysis, we conducted a Western blot to verify the expression of proteins related to the PI3K/Akt/Nrf2 pathway. The results showed that t-BHP decreased the phosphorylation of PI3K and Akt. In contrast, PSE pretreatment effectively activated PI3K and Akt and upregulated the expression of Nrf2, HO-1, and NQO1, thereby protecting HT-22 cells from oxidative stress damage induced by t-BHP. Overall, these findings provide compelling evidence for the central role of the PI3K/Akt/Nrf2 signaling axis in the neuroprotection mediated by PSE (Figure 6).

## 4. Materials and Methods

### 4.1. Materials

Peanut seeds (HuaYu No. 25) were harvested from Shandong province, China. Vanillin, proanthocyanidins, Folin and Ciocalteu’s phenol reagent, and rutin were purchased from Yuanye Bio-Technology (Shanghai, China). Gallic acid, ascorbic acid, iron (III), 2,2′-azino-bis (3-ethylbenzothiazoline-6-sulfonic acid) diammonium salt (ABTS), sodium acetate, and 2,4,6-tris (2-pyridyl)-s-triazine (TPTZ) were obtained from Macklin Biochemical Technology (Shanghai, China). DPPH free radical scavenging capacity assay kit was purchased from Nanjing Jiancheng Bioengineering Institute (Nanjing, China). One-step TUNEL apoptosis assay kit and mitochondrial membrane potential assay kit were products of Beyotime (Shanghai, China); CheKine™ Micro Superoxide Dismutases (SOD) Activity Assay Kit, CheKine™ Micro Lipid Peroxidation (MDA) Assay Kit, CheKine™ Micro Glutathione Oxidized (GSSG) Assay Kit, and CheKine™ Micro Reduced Glutathione (GSH) Assay Kit were products of Abbkine (Wuhan, China).

### 4.2. Preparation of PSE

Peanut skin powder was obtained by subjecting peanut seeds to repeated cycles of freezing and thawing, then grinding them to a powdered state. The powder was mixed with 95% ethanol (1:10, m/v) and ultrasonically extracted (40 KHz, 500 W) for 30 min at room temperature three times. Following this, the extracts were combined and centrifuged at 4000 rpm for 20 min. The resulting supernatants were then concentrated with a rotating vacuum evaporator at 40 °C and freeze-dried. The obtained freeze-dried samples were stored at −80 °C.

### 4.3. TPC, TFC, and TPAC Content Analysis

The TPC, TFC, and TPAC were quantified using the Folin–Ciocalteu [22], AlCl_3_ colorimetric [48], and Vanillin-HCl methods [49], respectively. The results of TPC, TFC, and TPAC were represented as mg GAE, RE, and PE/g peanut skin powder, respectively.

### 4.4. Chemical-Based Antioxidant Activity Assays

The DPPH and ABTS^+^ radical scavenging activities were determined using the ethanolic DPPH solution and the ABTS^+^ working solution, respectively [50]. The •OH-RSA was measured with the Fenton-type reaction method as described by Smirnoff and Cumbes [51]. The FRAP activity was quantified with the FRAP reagent, which comprises sodium acetate buffer (300 mmol/L, pH 3.6), TPTZ solution (10 mmol/L), and iron (III) chloride (20 mmol/L) at a ratio of 10:1:1 (*v*/*v*/*v*) [52]. The results of ABTS, FRAP, and •OH-RSA were expressed as mg AAE/g peanut skin powder, and DPPH was expressed as mg TE/g peanut skin powder.

### 4.5. Cell Culture and Treatment

HT-22 mouse hippocampal neuronal cells were obtained from the American Type Culture Collection (ATCC, VA, USA). They were cultured at 37 °C with the high glucose DMEM medium (Gibco, Gaithersburg, MD, USA) supplemented with 10% FBS (Gibco, Gaithersburg, MD, USA) and 1% penicillin–streptomycin (Gibco, Gaithersburg, MD, USA) in a humidified atmosphere with 5% CO_2_. The oxidative stress model was induced by treating cells with 300 μM tert-butyl hydroperoxide (t-BHP) (Sigma, St. Louis, MO, USA) for varying periods, as required by the specific assays. The cells were pretreated with different concentrations of PSE or 8.71 μg/mL µM Eda (positive control) for 24 h before being exposed to t-BHP damage.

### 4.6. Cell Viability Assay

Cell viability was evaluated using the Cell Counting Kit-8 (CCK-8, Dojindo Laboratories, Kumamoto, Japan) in accordance with the manufacturer’s instructions. Briefly, following a 24 h treatment with t-BHP, the cells were incubated with the CCK-8 solution in DMEM media (1:10) for 1 h. The absorbance was then measured at 450 nm. Cell viability was expressed as the percentage of the control group.

### 4.7. Detection of Cell Apoptosis

Apoptosis was analyzed using a one-step TUNEL apoptosis assay kit according to the manufacturer’s instructions. In brief, cells were fixed with 4% formaldehyde for 30 min after treatment with t-BHP for 4 h. Cells were then incubated with enhanced immunosuppressive permeabilization buffer for 5 min at room temperature, followed by staining with TdT-mediated dUTP nick-end labeling solution for 1 h at 37 °C in the dark. After DAPI staining (10 µg/mL for 5 min at room temperature), images were captured with a confocal laser scanning microscope (CLSM) (A1HD25, Nikon, Shanghai, China). The number of apoptotic cells (TUNEL-positive cells) and the total number of cells were counted using Image J software, and the apoptotic ratio was calculated as the number of apoptotic cells/total number of cells × 100%.

### 4.8. Detection of Intracellular ROS Levels

Intracellular ROS accumulation was detected using the fluorescent dye DCFH-DA. Briefly, after incubation with 300 μM t-BHP for 4 h, the cells were stained with 10 μM DCFH-DA for 20 min at 37 °C in the dark. Images were then captured by CLSM, and the fluorescence intensity was quantified using Image J software. ROS levels were expressed as the average fluorescence intensity of the treatment group relative to the control group.

### 4.9. Mitochondrial Membrane Potential (ΔΨ_m_) Assay

Mitochondrial membrane potential was detected using the JC-1 method. Briefly, after exposure to 300 μM t-BHP for 4 h, cells were stained with JC-1 staining working solution for 20 min at 37 °C in the dark. Images were then acquired using CLSM, and the intensity of the red and green fluorescence signals were quantified using Image J software. ΔΨm was expressed as the ratio of the intensity of the red signal to that of the green signal.

### 4.10. Measurement of SOD Activity and Levels of MDA, GSSG, and GSH

The detection kits for SOD, MDA, GSSG, and GSH (Abbkine Scientific Co., Ltd., Wuhan, China) were used to measure the intracellular levels of these markers. Briefly, the cells were harvested, lysed, and centrifuged to obtain the supernatant. The WST-1 approach was employed to assess the activity of SOD. MDA was quantified using the TBA (thiobarbituric acid) method. The DTNB (5,5′-dithiobis-(2-nitrobenzoic acid)) technique was employed for the detection of GSSG and GSH. In order to ensure comparability of the data, the protein concentrations of the supernatants were normalized using the BCA method.

### 4.11. Identification of the Potential Neuroprotective Targets of Phenolic Compounds in Peanut Skins

The major phenolic compounds in peanut skins were identified by a literature review [25,26,27]. Their SMILES structural formulae were obtained from the PubChem database, and the potential targets were predicted using SwissTargetPrediction software (http://swisstargetprediction.ch, accessed on 4 April 2025). Targets for “neuroprotection” were selected from the GeneCards, Online Mendelian Inheritance in Man database (OMIM), Therapeutic Target Database (TTD), DrugBank, and Pharmgkb databases. The overlapping targets between the target genes of phenolic compounds in peanut skins and neuroprotection were analyzed and visualized using Venny 2.1 online software (https://bioinfogp.cnb.csic.es/tools/venny/, accessed on 21 January 2025).

### 4.12. Construction of the PPI Network

The PPI network was constructed using the STRING database with overlapping targets at a confidence level of 0.7. The results were then imported into Cytoscape 3.9.1 software and visualized. The degree and betweenness centrality of the central network were further analyzed using the CytoNCA plugin.

### 4.13. GO and KEGG Enrichment Analysis

The GO and KEGG enrichment analyses were performed using the DAVID database with the overlapping targets. The top 20 terms in the GO analysis and the top 30 pathways in the KEGG analysis were then visualized as bar and bubble graphs using an online tool (https://www.bioinformatics.com.cn, accessed on 21 January 2025), respectively.

### 4.14. Western Blot Analysis

Cells were lysed with RIPA buffer (Beyotime, Shanghai, China) containing 1 mM PMSF (Beyotime, Shanghai, China) and phosphate inhibitor cocktails (Beyotime, Shanghai, China), and total proteins were collected. SDS-PAGE was used to separate the proteins (10 μg), which were then transferred to the polyvinylidene fluoride (PVDF) membrane (Bio-rad, Hercules, CA, USA). The membrane was blocked with 5% BSA (Beyotime, Shanghai, China) and incubated with the corresponding primary antibodies overnight at 4 °C. After being washed with TBST (Tris Buffered Saline + Tween-20), the membrane was then incubated with horseradish peroxidase-conjugated anti-rabbit secondary antibody (Cell Signaling Technology, Danvers, MA, USA), 1:3000) for 1 h at room temperature. Protein bands were visualized with an enhanced chemiluminescence (ECL) substrate kit (Bio-rad, Hercules, CA, USA) and analyzed using Image J software. The primary antibodies used in this research were as follows: Antibodies from Cell Signaling Technology (Danvers, MA, USA): Nrf2 (E5F1A, 1:1000), HO-1 (E3F4S, 1:1000), Cytochrome.c (D18C7, 1:000), pAkt (Ser473, 1:1000), Akt (9272, 1:1000), PI3K (4292, 1:1000), pPI3K (E3U1H, 1:1000), β-actin (D6A8, 1:3000), GAPDH (14C10, 1:3000), and anti-rabbit IgG (7074, 1:3000). An antibody from Abcam (Cambridge, UK): NQO1 (EPR3309, 1:1000).

### 4.15. Statistical Analysis

GraphPad Prism 5 (GraphPad Software, San Diego, California, USA) was used for all statistical analyses. Data were analyzed and presented as means ± standard deviation. For multiple group comparisons, one-way ANOVA was used, followed by Tukey’s test for pairwise comparisons. Significance was established at *p* < 0.05.

## 5. Conclusions

In conclusion, our study demonstrated that PSE exerted significant neuroprotective effects against oxidative damage in HT-22 cells. Cells pretreated with PSE maintained oxidative balance and were resistant to mitochondrial dysfunction and apoptotic cell death induced by the pro-oxidant t-BHP. The effects of PSE were attributed to its radical scavenging ability, as well as the enhanced endogenous antioxidant defense and mitochondrial integrity through activation of the PI3K/Akt/Nrf2 signaling axis. This study provides novel insights into the neuroprotective mechanisms of PSE and highlights its potential as a cost-effective, natural antioxidant source for the development of functional foods with neuroprotective properties.

## Figures and Tables

**Table 1:** The content of polyphenols in PSE and their antioxidant capacity in chemical-based systems.

**The Content of Polyphenols**	**TPC** **(mg GAE/g)**	**TFC** **(mg RE/g)**	**TPAC** **(mg PE/g)**	
	123.90 ± 0.46	75.97 ± 0.23	53.34 ± 1.58	
**Antioxidant capacity**	**ABTS** **(mg AAE/g)**	**DPPH** **(mg TE/g)**	**•OH-RSA** **(mg AAE/g)**	**FRAP** **(mg AAE/g)**
	230.36 ± 15.96	10.21 ± 0.12	136.49 ± 3.26	14.59 ± 1.12

TPC: total phenolic content; TFC: total flavonoid content; TPAC: total proanthocyanidin content; GAE: gallic acid equivalent; RE: rutin equivalent; PE: proanthocyanidin equivalent; ABTS: 2,2′-azino-bis (3-ethylbenzothiazoline-6-sulfonic acid) diammonium salt; DPPH: 2,2-diphenyl-1-picrylhydrazyl; •OH-RSA: hydroxyl radical scavenging activity; FRAP: ferric reducing antioxidant power; TE: Trolox equivalent; AAE: ascorbic acid equivalent.

**Table 2:** Major phenolic compounds in peanut skins.

Phenolic Acids	Flavonoids	Stilbenes
Caffeic acid	Catechin	Resveratrol
p-Coumaric acid	Epigallocatechin gallate	Piceatannol
Ellagic acid	Rutin	Piceid
Chicoric acid	Epigallocatechin	
Ferulic acid	Naringenin	
Gallic acid	Catechin gallate	
	Eriodictyol	
	proanthocyanidin	

## Data Availability

The data presented in this study are available on request from the corresponding author. The data are not publicly available to protect potential patentable aspects of the research.

## Abbreviations

•OH-RSA, radical scavenging activity; AAE, ascorbic acid equivalent; ABTS, 2,2′-azino-bis (3-ethylbenzothiazoline-6-sulfonic acid) diammonium salt; AKT1, AKT serine/threonine kinase 1; CCK-8, cell counting kit-8; CLSM, confocal laser scanning microscope; CNS, central nervous system; DTNB, (5,5′-dithiobis-(2-nitrobenzoic acid)); Eda, edaravone; ETC, electron transport chain; FRAP, ferric reducing antioxidant power; GAE, gallic acid equivalent; GO, gene ontology; GSH, glutathione; GSSG, oxidized glutathione; HO-1, heme oxygenase-1; KEGG, Kyoto Encyclopedia of Genes and Genomes; MDA, malondialdehyde; NQO1, NAD(P)H dehydrogenase quinone 1; Nrf2, nuclear factor erythroid 2-related factor 2; OXPHOS, oxidative phosphorylation; PE, proanthocyanidin equivalent; PI3K/Akt, phosphatidylinositol 3-kinase/protein kinase B; PPI, protein–protein interaction; PS, peanut skin; PSE, peanut skin extract; RE, rutin equivalent; ROS, reactive oxygen species; SOD, superoxide dismutase; t-BHP, t-butyl hydroperoxide; TE, Trolox equivalent; TFC, total flavonoid content; TPAC, total proanthocyanidin content; TPC, total phenolic content; TPTZ, 2,4,6-tris (2-pyridyl)-s-triazine.
